# Picturing Breast Cancer Brain Metastasis Development to Unravel Molecular Players and Cellular Crosstalk

**DOI:** 10.3390/cancers13040910

**Published:** 2021-02-22

**Authors:** Inês Figueira, Sofia Galego, Tânia Custódio-Santos, Raquel Vicente, Kinga Molnár, Janos Haskó, Rui Malhó, Mafalda Videira, Imola Wilhelm, István Krizbai, Maria Alexandra Brito

**Affiliations:** 1 Farm-ID—Associação da Faculdade de Farmácia para a Investigação e Desenvolvimento, 1649-003 Lisbon, Portugal; ifigueira@farm-id.pt; 2 Research Institute for Medicines (iMed.ULisboa), Faculty of Pharmacy, Universidade de Lisboa, 1649-003 Lisbon, Portugal; sofiagalego11@hotmail.com (S.G.); taniacustodiosantos@gmail.com (T.C.-S.); raquelhvicente@gmail.com (R.V.); mvideira@ff.ulisboa.pt (M.V.); 3 Institute of Biophysics, Biological Research Centre, Eötvös Loránd Research Network (ELKH), 6726 Szeged, Hungary; molnar.kinga@brc.hu (K.M.); hasko.janos@brc.hu (J.H.); wilhelm.imola@brc.hu (I.W.); krizbai.istvan@brc.hu (I.K.); 4 BioISI, BioSystems and Integrative Sciences Institute, Faculty of Sciences, Universidade de Lisboa, 1749-016 Lisbon, Portugal; rmmalho@fc.ul.pt; 5 Department of Pharmacy, Pharmacology and Health Technologies, Faculty of Pharmacy, Universidade de Lisboa, 1649-003 Lisbon, Portugal; 6 Institute of Life Sciences, Vasile Goldis Western University of Arad, 310025 Arad, Romania; 7 Department of Medicine and Pharmaceutical Sciences, Faculty of Pharmacy, Universidade de Lisboa, 1649-003 Lisbon, Portugal

**Keywords:** blood–brain barrier, breast cancer brain metastasis, extravasation, intercellular communication, mesenchymal–epithelial transition, microvasculature

## Abstract

**Simple Summary:**

Breast cancer is a devastating disorder affecting millions of women worldwide. With improved therapeutics for the primary tumor, the appearance of metastasis has been increasing. Breast cancer frequently metastasizes to the brain, constituting a major hurdle without cure and with a poor survival. It is imperative to better understand the mechanisms involved in malignant cell transposition of the brain microvasculature and parenchymal colonization by deciphering the alterations occurring in the tumor and microvascular cells, as well as the occurrence of intercellular communication during the process. We aimed to profile the process of the formation of breast cancer brain metastasis and the timeline of events governing it. We used a specific mouse model of the disease to perform extensive microscopic analyses. We identified phenotypic changes and the activation of relevant molecular players in tumorigenesis, together with vascular alterations, and the occurrence of crosstalk. Our findings unravel putative therapeutic targets to tackle breast cancer brain metastasis.

**Abstract:**

With breast cancer (BC) therapy improvements, the appearance of brain metastases has been increasing, representing a life-threatening condition. Brain metastasis formation involves BC cell (BCC) extravasation across the blood–brain barrier (BBB) and brain colonization by unclear mechanisms. We aimed to disclose the actors involved in BC brain metastasis formation, focusing on BCCs’ phenotype, growth factor expression, and signaling pathway activation, correlating with BBB alterations and intercellular communication. Hippocampi of female mice inoculated with 4T1 BCCs were examined over time by hematoxylin-eosin, immunohistochemistry and immunofluorescence. Well-established metastases were observed at seven days, increasing thereafter. BCCs entering brain parenchyma presented mesenchymal, migratory, and proliferative features; however, with time, they increasingly expressed epithelial markers, reflecting a mesenchymal–epithelial transition. BCCs also expressed platelet-derived growth factor-B, β_4_ integrin, and focal adhesion kinase, suggesting autocrine and/or paracrine regulation with adhesion signaling activation, while balance between Rac1 and RhoA was associated with the motility status. Intercellular communication via gap junctions was clear among BCCs, and between BCCs and endothelial cells. Thrombin accumulation, junctional protein impairment, and vesicular proteins increase reflect BBB alterations related with extravasation. Expression of plasmalemma vesicle-associated protein was increased in BCCs, along with augmented vascularization, whereas pericyte contraction indicated mural cells’ activation. Our results provide further understanding of BC brain metastasis formation, disclosing potential therapeutic targets.

## 1. Introduction

Breast cancer (BC) represents the leading cause of neoplastic disease in women, with an incidence of more than two million new cases and more than 620,000 deaths estimated in 2018 [1]. The remarkable advances in novel therapies have improved BC patients’ life quality and survival, rendering development of distant organ metastases a major concern. Brain metastases occur in approximately 15% of BC patients [2], with the highest incidences occurring in human epidermal growth receptor (HER) 2-positive and triple-negative (TN) BC (TNBC) [3]. Therefore, BC brain metastases (BCBM) represent a massive and devastating problem, with a survival rate of only 20% one year after diagnosis [4]. To this appalling scenario accounts the fact that the cellular and molecular events occurring during extravasation across the blood–brain barrier (BBB) and brain colonization are still marginally understood, together with the lack of efficient therapies for this secondary tumor.

Establishment of BCBM is a complex process comprising several steps, such as the invasion of the mammary tissue by BC cells (BCCs), their entrance into blood vessels (intravasation), their arrest, attachment and migration across endothelial cells (ECs) of brain microvasculature (extravasation), and the colonization of the brain parenchyma [4]. During these events, BCCs undergo phenotypic changes that determine their metastatic profile. In the primary tumor, BCCs lose epithelial proteins such as lectin and cytokeratin and gain mesenchymal proteins such as vimentin and neuronal (N)-cadherin, a process known as epithelial–mesenchymal transition (EMT), which endows cells with invasive and migratory properties. Once in the brain, they lose the mesenchymal proteins and reacquire the epithelial proteins, known as mesenchymal–epithelial transition (MET), which favors BCBM development [4]. Moreover, BCCs colonizing the brain express vascular endothelial growth factor (VEGF), which is involved in new vessel formation to allow an optimal oxygen supply and access to nutrients, favoring metastasis development [5], as well as other determinant growth factors including platelet-derived growth factor B (PDGF-B) [6].

Development of BCBM involves cytoskeleton-associated alterations and activation of signaling pathways that regulate adhesion, migration, and invasion. For instance, focal adhesion kinase (FAK) has a central role in adhesion, survival, proliferation, invasion, angiogenesis, and metastases formation through the activation of integrin signaling [7], which has been associated with tumor progression, namely in invasive and metastatic processes [8]. In fact, it was demonstrated that the β_4_ integrin subunit (part of the α_6_β_4_ integrin dimer) interacts with FAK, forming β_4_ integrin/FAK complexes via AKT signaling, which in turn promotes angiogenesis, cancer cell invasion and anchorage-independent growth [9]. Moreover, the Rho family of small GTPases regulates cytoskeleton and actomyosin contractility, playing an important role in motility and tumor cell extravasation [10,11]. The effector myosin light chain kinase (MLCK) mediates perijunctional apical actomyosin ring contraction, leading to cytoskeleton rearrangement that modulates vascular functions associated with new vessel formation [12,13]. However, the involvement of such events in BCBM formation and their temporal interplay remain undetermined.

To successfully extravasate into the brain, tumor cells must face and overcome the protective and complex structure known as the BBB [14]. The BBB microvasculature is characterized by elaborate junctional complexes, including tight and adherens junctions (TJs and AJs, respectively) that act to restrict the permeability across the endothelium, as well as gap junctions (GJs) that mediate intercellular communication [15]. The interaction between malignant cells and brain microvascular ECs (BMECs) was shown to induce a redistribution and reorganization of junctional and cytoskeleton proteins, with junctions’ opening that allows the squeeze of BCCs between two BMECs (paracellular transmigration) and can lead to BBB disruption [16,17]. We have demonstrated that BCCs are incorporated into the endothelium and migrate through the transcellular way [18], pointing to different possible transmigration mechanisms. Brain capillaries are ensheathed by pericytes, which are mural cells with contractile properties that play a crucial role in the regulation of microcirculation and maintenance of BBB permeability [19], but their contribution to extravasation and BCBM formation is poorly explored. We have also shown that pericytes play a crucial role in the development of metastatic brain tumors by directly influencing key steps of disease progression, including the adhesion, migration, and proliferation of TNBC cells [20]. Furthermore, the crosstalk between malignant and brain resident cells was shown to favor brain metastases formation [21]. As such, BCCs communication with brain cells and among them will certainly influence disease progression and spread, thus deserving to be further investigated. 

Neovascularization, a complex process involving extracellular matrix (ECM) remodeling, EC migration, proliferation, and re-differentiation into more mature vessels, has been described as one of the mechanisms necessary for optimal tumor growth [22]. Tumor angiogenesis in particular has been associated with increased levels of plasmalemma vesicle-associated protein (PLVAP), being typically induced in ECs of large, well-vascularized tumors [23]. PLVAP forms the diaphragms of caveolae, fenestrae and trans-endothelial channels in ECs, being expressed in pathological conditions associated with compromised barrier function such as cancer [24]. However, the expression of this protein in BCBM remains undetermined.

Being aware of all the unanswered questions regarding BCC extravasation and brain colonization, we aimed to understand and characterize the in vivo profile of BCBM formation. We unraveled malignant cell phenotype alterations and major signaling pathways involved during the process. Cell crosstalk, pericyte activation, and increased vascularization roles for a successful brain colonization were revealed. TJ and AJ alterations, caveolin-1 expression, and blood-borne thrombin accumulation in the brain were also observed, reflecting both paracellular and transcellular alterations along extravasation, and BBB disruption. By establishing the temporal sequence of events in BCBM formation and by identifying the early ones, this study contributes to an in-depth understanding of the disease complexity and points to potential therapeutic targets for modulation to prevent BCBM development.

## 2. Results

### 2.1. 4T1 Cell Injection Leads to the Establishment of Hippocampal BCBM with PDGF-B and Ki-67-Positive Cells

To establish the temporal evolution of BCBM development and the actors involved in the process, we took advantage of an established mouse model. The TN 4T1 cells, one of the most aggressive BCCs [25], were inoculated in the common carotid artery to direct the malignant cells to the brain and allow preferential brain metastases formation [26]. Considering that the most prominent metastases development was observed in the cranial hippocampus, this was the brain region analyzed in the present study.

**Figure 1:**
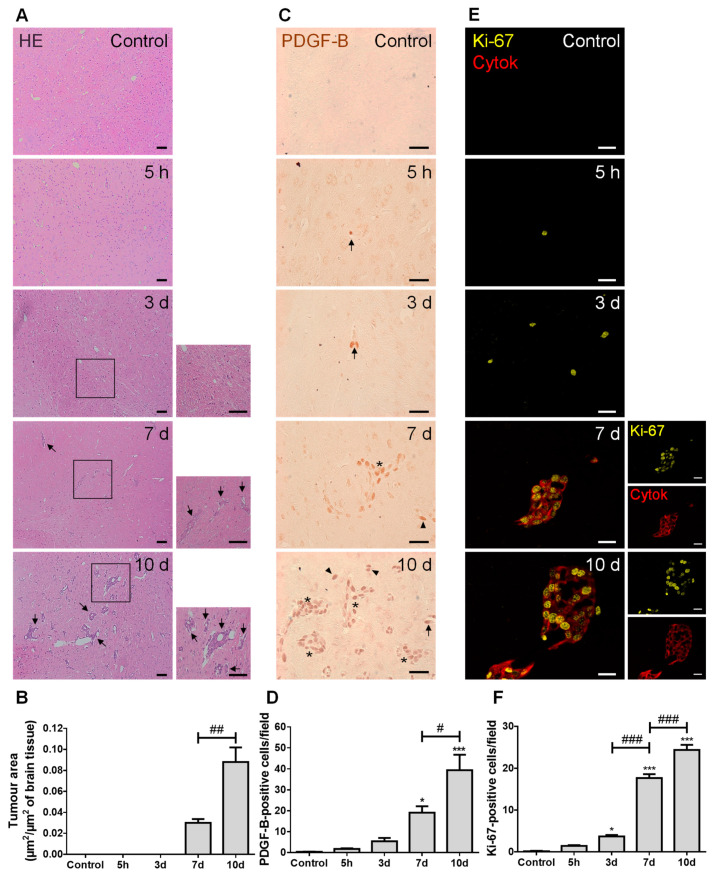
Injection of triple negative (TN) breast cancer cells (BCCs) leads to a time-dependent formation of metastases with PDGF-B and Ki-67-positive cells. Either 4T1 cells or vehicle (controls) were inoculated in the carotid arteries of female Balb/c mice and hippocampal sections were analyzed after 5 h (h), 3, 7 and 10 days (d). (**A**) The pattern of breast cancer brain metastases formation assessed by hematoxylin-eosin (HE) staining revealed well-established metastases from 7 d onwards (black arrows). Scale bar: 40 µm. Magnification of the areas within the squares is shown. (**B**) Semi-quantitative analysis of the tumor area revealed an increase over time. (**C**) PDGF-B expression was observed in BCCs (brownish staining) inside blood vessels (arrows), in scattered cells (arrowheads) or in metastases surrounding blood vessels (asterisks). Scale bar: 20 µm. (**D**) Quantification of the number of PDGF-B-positive cells revealed an increase over time. (**E**) Ki-67-positive (yellow) BCCs were observed in cytokeratin (cytok)-positive (red) metastases. Scale bar: 20 µm. (**F**) Quantification of the number of Ki-67-positive cells revealed an increase over time. Statistical differences are denoted as * *p* < 0.05, *** *p* < 0.001 vs. control, and # *p* < 0.05, ## *p* < 0.01, ### *p* < 0.001 between indicated groups. Data are mean ± SEM, *n* = 6.

We started by characterizing the metastases development pattern (Figure 1). In line with our previous study [26], well-established BCBMs were observed at seven days and augmented thereafter (Figure 1A), with a circa three-fold increase from 7 to 10 days (Figure 1B). PDGF-B, a growth factor described to be upregulated in brain tumors [27], was predominantly expressed by BCCs located near blood vessels, rather than by BMECs (Figure 1C). Importantly, as early as at five hours after BCC inoculation, PDGF-B-positive cells were already visible, even inside blood vessels before reaching brain parenchyma, which increased over metastasis development (Figure 1D). Aiming to understand if an increased tumor area over time resulted from an increased proliferative capacity of BCCs, we assessed the expression of the proliferation marker, Ki-67, together with the epithelial marker, pan cytokeratin, in order to visualize metastatic lesions. A clear proliferative phenotype (Ki-67-positive cells) was observed at as early as five hours, being particularly evident in pan cytokeratin-positive cells, especially in the periphery of metastases (Figure 1E). Importantly, the number of Ki-67-positive cells significantly increased with time (Figure 1F), reflecting tumor cells’ proliferation during disease progression. 

### 2.2. Malignant Cells Undergo a Mesenchymal–Epithelial Transition (MET)

**Figure 2:**
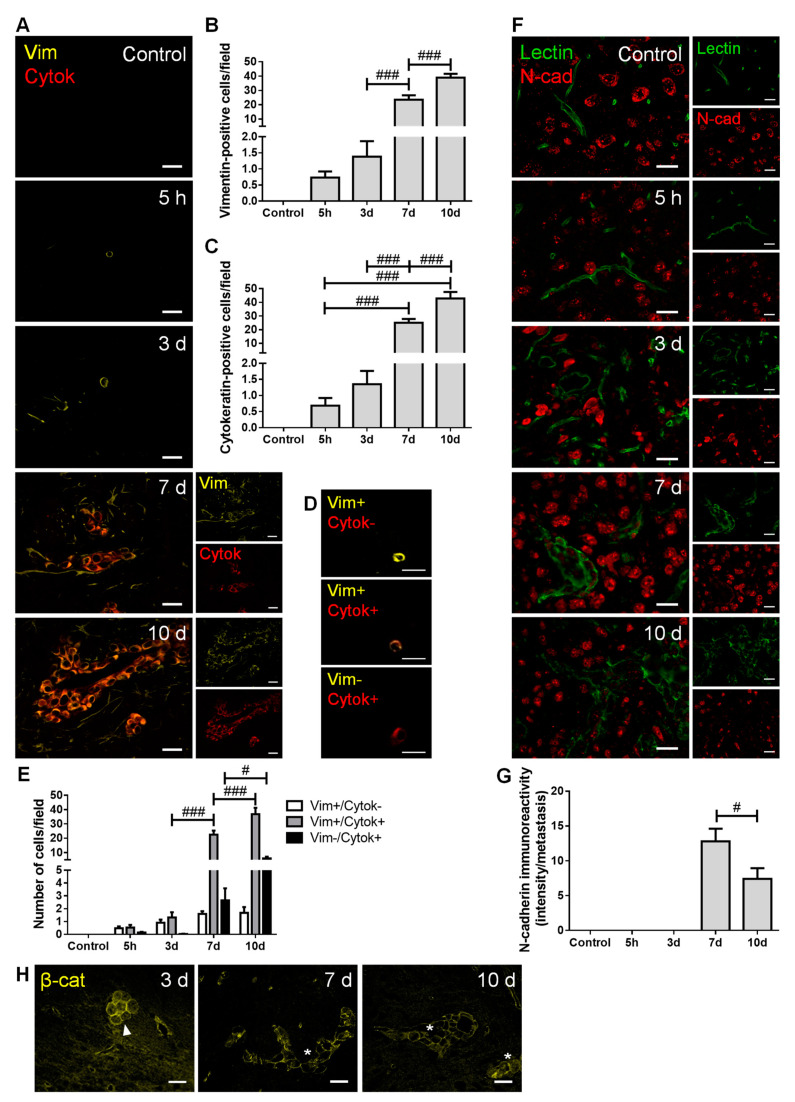
Breast cancer cells (BCCs) undergo a mesenchymal to epithelial transition. The 4T1 cells or vehicle (control) were inoculated in the carotid arteries of female Balb/c mice and hippocampal sections were analyzed after 5 h (h), 3, 7 and 10 days (d). (**A**) Double labelling with vimentin (vim, yellow, mesenchymal) and cytokeratin (cytok, red, epithelial) showed vimentin-positive cells entering brain parenchyma, leading to the formation of vimentin-positive and cytokeratin-positive metastases. Scale bar: 20 µm. Semi-quantitative analysis of vimentin-positive and cytokeratin-positive cells (**B**,**C**, respectively) revealed an increase in both markers in well-established metastases. (**D**) Different tumor cell subpopulation phenotypes were observed: vimentin-positive/cytokeratin-negative (vim+/cytok-), vimentin-positive/cytokeratin-positive (vim+/cytok+), and vimentin-negative/cytokeratin-positive (vim-/cytok+). Scale bar: 20 µm. (**E**) Quantitative analysis of the cell subpopulations per field revealed that most cells express both markers. (**F**) Double labelling with N-cadherin (N-cad, red, mesenchymal) and tomato lectin (green, epithelial) showed brain-resident N-cadherin-positive cells, lectin-positive blood vessels, as well as N-cadherin-positive BCCs in lectin-positive metastases. Scale bar: 20 µm. (**G**) Semi-quantitative analysis of N-cadherin in metastases revealed a significant decrease at 10 d. (**H**) β-catenin (β-cat, yellow) expression was observed in metastatic cells (3 d, arrowhead) and in well-established metastases (7 and 10 d, asterisk). Scale bar: 20 µm. Statistical differences are denoted as # *p* < 0.05 and ### *p* < 0.001 between indicated groups. Data are mean ± SEM, *n* ≥ 3.

To establish BCCs’ phenotype along brain metastases development, the expression of mesenchymal (vimentin and N-cadherin) and epithelial markers (pan cytokeratin, tomato lectin and β-catenin) was assessed (Figure 2). 

We observed that BCCs entering and colonizing the brain parenchyma express vimentin (Figure 2A), reflecting a mesenchymal phenotype determinant for cell migration. The number of vimentin-positive cells increased throughout metastatic development (Figure 2B), as the malignant cells populating the parenchyma increased. Moreover, the number of cytokeratin-positive cells also increased with metastases development (Figure 2C), indicating that BCCs increasingly express epithelial features. Interestingly, BCCs at the metastasis periphery predominantly express vimentin, compatible with a mesenchymal phenotype that favors further cell migration and spread. For an in-depth analysis, malignant cells were classified into three groups, according to their phenotype: vimentin-positive/cytokeratin-negative, vimentin-positive/cytokeratin-positive, and vimentin-negative/cytokeratin-positive (Figure 2D). The semi-quantitative analysis showed that the vimentin-positive/cytokeratin-negative cells was the smallest population, whereas the vimentin-positive/cytokeratin-positive cells comprised the largest one. Moreover, the vimentin-positive/cytokeratin-positive phenotype increased from three to seven days and continued to increase afterwards. An augment of vimentin-negative/cytokeratin-positive cells was also observed, although not as marked as that of vimentin-positive/cytokeratin-positive phenotype (Figure 2E). 

Analysis of N-cadherin revealed its clear expression in resident brain cells, as well as in well-established BCBM at 7 and 10 days (Figure 2F). To assess N-cadherin expression in malignant cells, while assuring that parenchymal cells were not considered, N-cadherin labelling intensity was assessed in well-established metastases identified by the epithelial marker tomato lectin. As shown in Figure 2C, cells within lesions express the mesenchymal marker, of which the intensity decreases as disease progresses (*p* < 0.05 between 7 and 10 days). These results point to a reduction in mesenchymal features in larger metastasis, accompanied by the appearance of tomato lectin-positive cells that reinforce the development of epithelial characteristics by malignant cells. Parallelly, we observed that early metastatic cells (three days) express β-catenin, which presents a preferential membrane localization as metastases develop (Figure 2H), a reflex of epithelial characteristic acquisition. 

These results indicate an upregulation of epithelial markers in BCCs as metastases development progresses, suggesting the occurrence of MET, although some mesenchymal features are still maintained.

### 2.3. Adhesion and Migration-Associated Signaling Molecules Are Involved in BCBM Formation

**Figure 3:**
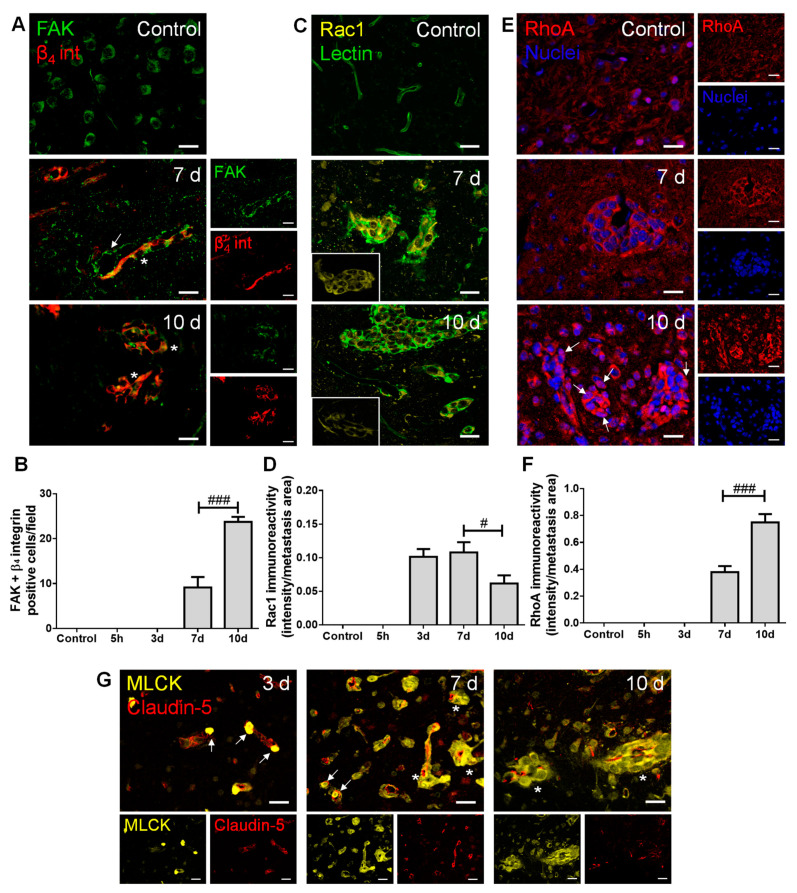
Adhesion and migration-associated signaling molecules are expressed in breast cancer (BC) brain metastases. The 4T1 cells or vehicle (controls) were inoculated in the carotid arteries of female Balb/c mice and hippocampal sections were analyzed after 5 h (h), 3, 7 and 10 days (d). (**A**) Double labelling with focal adhesion kinase (FAK, green) and β_4_ integrin (β_4_ int, red) showed brain-resident FAK-positive cells, FAK-positive blood vessels feeding metastases (arrow) and co-labelling of FAK and β_4_ integrin in metastatic lesions (asterisks). Scale bar: 20 µm. (**B**) Quantification of co-labelled FAK and β_4_ integrin (FAK + β_4_ integrin)-positive cells per field revealed an increase from 7 to 10 d. (**C**) Ras-related C3 botulinum toxin substrate 1 (Rac1)-positive (yellow) BC cells were observed in lectin-positive (green) brain metastasis, which peaked at 7 d (magnified sections). Scale bar: 20 µm. (**D**) Semi-quantitative analysis revealed that Rac1 decreases from 7 to 10 d. (**E**) RhoA-positive (red) BC cells were observed in well-established metastasis, with malignant cells presenting nuclear translocation at 10 d (arrows). Nuclei (blue) were counterstained with Hoechst 33342. Scale bar: 20 µm. (**F**) Semi-quantitative analysis of RhoA immunoreactivity revealed a significant increase at 10 d. (**G**) Myosin light chain kinase (MLCK) (yellow) expression was observed in BC cells inside claudin-5-positive (red) blood vessels (arrows) and in formed metastases surrounding blood vessels (asterisks). Scale bar: 20 µm. Statistical differences are denoted as # *p* < 0.05, ### *p* < 0.001 between indicated groups. Data are mean ± SEM, *n* ≥ 3.

To establish the role of adhesion, invasion and migration-associated molecules in the metastatic process, we inspected the brain parenchyma for the presence of FAK and β4 integrin, as well as of Ras-related C3 botulinum toxin substrate 1 (Rac1), Ras homolog gene family member A (RhoA) and MLCK (Figure 3).

Regarding adhesion and integrin signaling, we observed that BCCs express both FAK and β4 integrin (Figure 3A), and that FAK and β_4_ integrin-positive cells increased in metastases from 7 to 10 days (Figure 3B), pointing to a possible signaling involving these two proteins in BCBM formation. Importantly, FAK expression was notorious in blood vessels at seven days, stressing its role at BBB endothelial level in metastases formation. This kinase has also been described in neural development and function [28], which corroborates our observations of FAK expression in brain cells resembling the morphology of neurons’ cell bodies in the control.

Actors in cell migration and the cytoskeleton remodeling network were also evaluated. We observed that the expression of the protein involved in mesenchymal-like migration, Rac1, appeared in metastasizing BCCs extravasating into the parenchyma and in established metastases (Figure 3C), of which the immunoreactivity per metastases area decreased after metastasis establishment (Figure 3D). Parallelly, RhoA expression was evident in well-established metastases (Figure 3E), an expression that significantly increased at 10 days (Figure 3F), reflecting a Rac1/RhoA balance. Interestingly, RhoA nuclear translocation was observed as a late event (Figure 3E, 10 days). MLCK expression was also quite evident in metastatic BCCs (three days onwards) in the vicinity of BMECs and still inside blood vessels (Figure 3G), pointing to a role of cytoskeleton contraction via MLCK in the establishment of BCBM.

### 2.4. Cellular Crosstalk in BCBM Occurs via Gap Junctions

**Figure 4:**
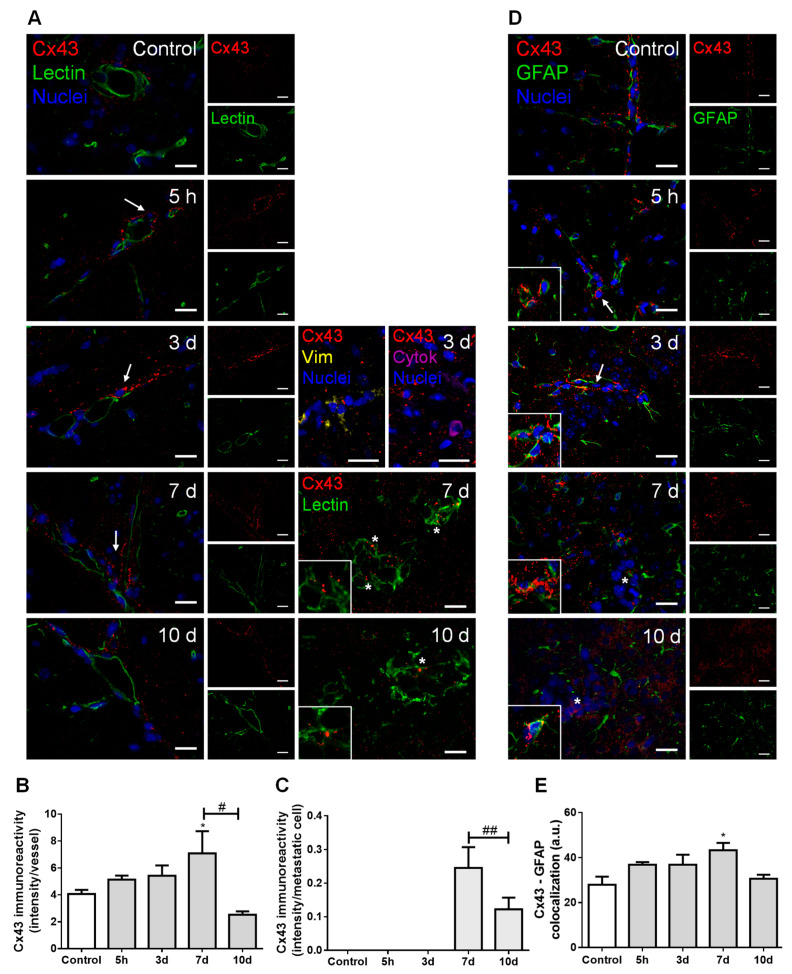
Cell crosstalk in breast cancer (BC) brain metastases occurs via gap junctions. The 4T1 cells or vehicle (controls) were inoculated in the carotid arteries of female Balb/c mice and hippocampal sections were analyzed after 5 h (h), 3, 7 and 10 days (d). (**A**) Double labelling of connexin 43 (Cx43, red) and the epithelial marker tomato lectin (green) showed that Cx43 is expressed in contact areas between endothelial cells and BC cells (arrows), in the vicinity of vimentin (yellow)- and cytokeratin (purple)-positive BC cells, as well as between adjacent BC cells (asterisks and magnified squares). Nuclei (blue) were counterstained with Hoechst 33342. Scale bar: 20 µm. Semi-quantitative analysis of Cx43 immunoreactivity per vessel (**B**) and per metastasis (**C**) revealed a peak at 7 d. (**D**) Double labelling of Cx43 (red) and the astrocyte marker glial fibrillary acidic protein (GFAP, green) showed the expression of this gap junction protein in contact areas among astrocytes (magnified squares) and between astrocytes and BC cells (arrows) but more distant from metastatic lesions (asterisk). Nuclei (blue) were counterstained with Hoechst 33342. Scale bar: 20 µm. (**E**) Semi-quantitative analysis of Cx43–GFAP colocalization revealed a peak at 7 d. Statistical differences are denoted as * *p* < 0.05 vs. control and # *p* < 0.05, ## *p* < 0.01 between indicated groups. Data are mean ± SEM, *n* = 3.

In order to understand the possible crosstalk between BCCs and brain resident cells along BCBM development, we inspected the expression of connexin 43 (Cx43), further characterizing the cell types expressing this junctional protein based on the epithelial marker tomato lectin and the astrocytic marker glial fibrillary acidic protein (GFAP) (Figure 4). 

Double labelling for Cx43 and tomato lectin showed Cx43 expression not only in the normal brain parenchyma (control), but also along metastases development (Figure 4A). Cx43 was observed in contact areas between BMECs and BCCs, namely vimentin-positive and cytokeratin-positive malignant cells, which have migrated across blood vessels, as well as between adjacent BCCs in well-established metastases. Semi-quantitative analysis of Cx43 intensity in blood vessels-associated areas and in well-established metastases showed that it peaks at seven days (Figure 4B,C, respectively). 

Hippocampal sections observation also revealed a widespread parenchymal Cx43 presence, suggesting its expression by astrocytes, known to be connected to each other by GJ composed of connexins such as Cx43 [29]. We observed a higher Cx43 expression in GFAP-expressing cells localized further from metastases than those closer to the tumor (Figure 4D). Moreover, a colocalization between these two proteins was observed, which peaked at seven days (Figure 4E), pointing to astrocyte recruitment and increased GJ immunoreactivity upon BCCs extravasation. 

### 2.5. Establishment of BCBM Leads to Increased Vascularization and BBB Disruption

**Figure 5:**
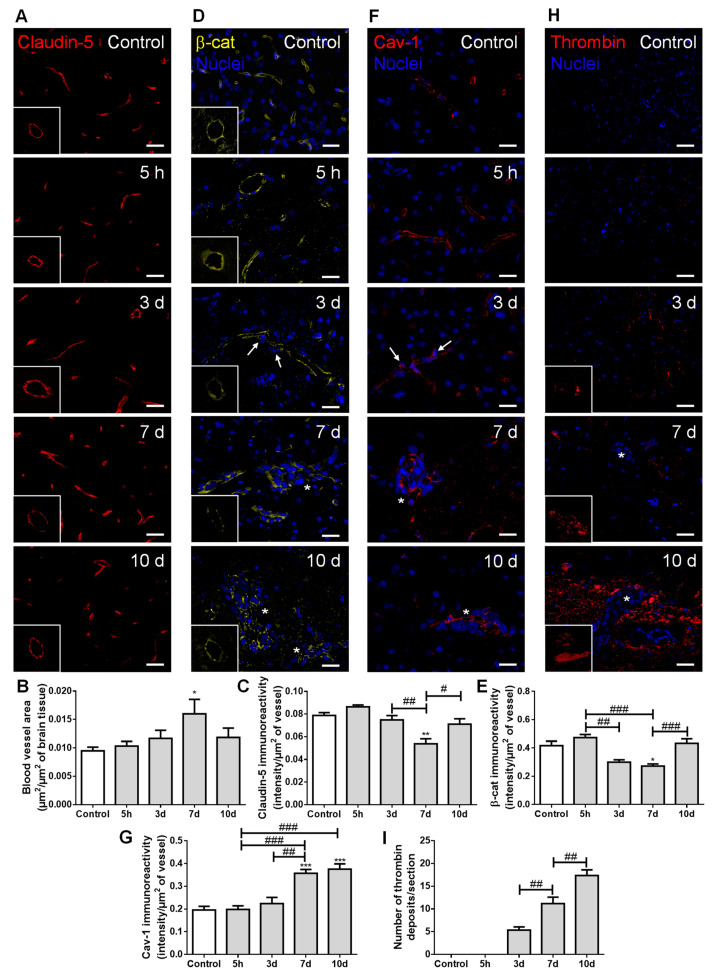
Establishment of breast cancer (BC) brain metastases lead to increased vascularization and blood–brain barrier disruption. The 4T1 cells or vehicle (controls) were inoculated in the carotid arteries of female Balb/c mice and hippocampal sections were analyzed after 5 h (h), 3, 7 and 10 days (d). (**A**) Claudin-5 (red) expression was observed at the brain microvasculature, where discontinuous labelling was observed at 7 d (squares with a representative transversal cut of a blood vessel). Scale bar: 20 µm. Semi-quantitative analysis revealed (**B**) increased vascularization and (**C**) decreased claudin-5 immunoreactivity per vessel. (**D**) β-catenin (β-cat, yellow) expression was observed at the brain microvasculature, diminishing at 3 and 7 d (squares with a representative transversal cut of a blood vessel). Nuclei (blue) were counterstained with Hoechst 33342. Scale bar: 20 µm. (**E**) Semi-quantitative analysis revealed a decrease in β-catenin immunoreactivity per vessel. (**F**) Vesicular transport protein caveolin-1 (Cav-1, red) expression was observed at the brain microvasculature, especially in blood vessels close to BC cells (arrows) and in well-established metastasis (asterisks). Nuclei (blue) were counterstained with Hoechst 33342. Scale bar: 20 µm. (**G**) Semi-quantitative analysis revealed an increase in caveolin-1 immunoreactivity per vessel. (**H**) Blood-borne thrombin (red) deposits were observed in the brain parenchyma, in the vicinity of metastases, increasing in size with time (magnified sections). Nuclei (blue) were counterstained with Hoechst 33342. Scale bar: 20 µm. (**I**) Semi-quantitative analysis revealed an increase in the number of thrombin deposits. Statistical differences are denoted as * *p* < 0.05, ** *p* < 0.01, *** *p* < 0.001 vs. control and # *p* < 0.05, ## *p* < 0.01, ### *p* < 0.001 between indicated groups. Data are mean ± SEM, *n* = 6.

To evaluate whether extravasation of BCCs into the brain and establishment of metastases are associated with increased vascularization and BBB disruption, we assessed the expression of claudin-5, β-catenin, caveolin-1, and thrombin (Figure 5).

Claudin-5-labelled sections allowed microvessels’ density visualization along metastases development (Figure 5A), which revealed an increase in vascularization, peaking at seven days (Figure 5B). This result points to the association of hypervascularization with brain colonization by BCCs. As expected, claudin-5 presented a normal distribution forming a continuous line at the cell–cell contacts in controls; however, as the metastatic disease progressed, this TJ protein showed an irregular and discontinuous distribution (Figure 5A). A significant decrease in claudin-5 immunoreactivity over time was observed, with the lowest expression occurring at seven days, tending to recover afterwards (Figure 5C). In accordance with such observations, the immunolabelling of the AJ protein β-catenin presented a dotted pattern at three days concomitant with extravasating cells (Figure 5D). This translated into a significant decrease in β-catenin intensity per vessel area from five hours to three and seven days after BCCs injection, with a partial recovery at 10 days (Figure 5E), similarly to claudin-5. 

Regarding transcellular permeability alterations, we observed that the transcytosis associated-protein, caveolin-1, was expressed at the microvasculature in the controls, being upregulated in blood vessels associated with metastases development (Figure 5F). Accordingly, a sustained increase in caveolin-1 immunoreactivity per vessel was observed from 3 to 7–10 days post-inoculation of BCCs (Figure 5G).

We also investigated if the paracellular and transcellular permeability alterations were accompanied by the entrance of thrombin into the brain parenchyma, an indicator of BBB disruption [30]. Small thrombin deposits were already evident at three days post-tumor cell injection, and an increase in deposit size was observed from three to seven days, further increasing until 10 days, especially in the proximity of metastatic lesions (Figure 5H). Accordingly, a progressive increase in the number of thrombin deposits was observed (Figure 5I). 

Altogether, these findings suggest increased BBB permeability, with both TJ and AJ disruption, as well as vesicular transport activation during BCCs’ extravasation and metastases establishment. Although some recovery of the junctional proteins’ expression occurred, it was not enough to prevent the thrombin accumulation in brain parenchyma during the enlargement of metastases.

### 2.6. Pericytes Are Involved in the Formation of BCBMs

**Figure 6:**
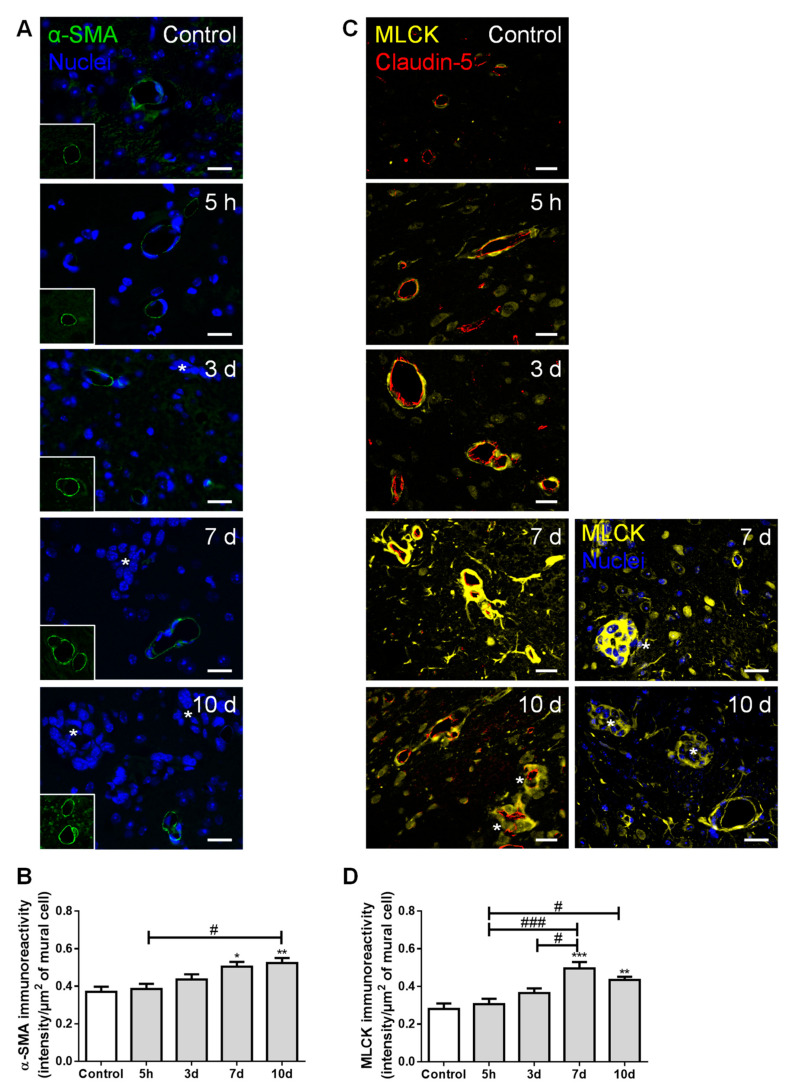
Formation of breast cancer brain metastases involves mural cells activation. The 4T1 cells or vehicle (controls) were inoculated in the carotid arteries of female Balb/c mice and hippocampal sections were analyzed after 5 h (h), 3, 7 and 10 days (d). (**A**) α-smooth muscle actin (α-SMA, green) protein expression was observed in mural cells, surrounding blood vessels. Scale bar: 20 µm. (**B**) Semi-quantitative analysis revealed a significant increase in α-SMA immunoreactivity per mural cell with time. (**C**) Myosin light chain kinase (MLCK, yellow) protein expression was observed in mural cells, surrounding claudin-5 positive (red) blood vessels. Scale bar: 20 µm. (**D**) Semi-quantitative analysis revealed a sustained increase in MLCK immunoreactivity per mural cell, from 7 d onwards. Metastatic lesions are indicated with asterisk. Nuclei (blue) were counterstained with Hoechst 33342. Statistical differences are denoted as * *p* < 0.05, ** *p* < 0.01, *** *p* < 0.001 vs. control and # *p* < 0.05, ### *p* < 0.001 between indicated groups. Data are mean ± SEM, *n* ≥ 4.

To ascertain mural cells’ (pericytes and smooth muscle cells) role regarding brain BCCs extravasation and BCBM formation, their specific markers, α-smooth muscle actin (α-SMA) and MLCK, were evaluated (Figure 6). 

We observed the expression of α-SMA, a protein required for mural cells’ contraction [31], namely around blood vessels and near metastatic cells (Figure 6A). We further observed a significant increase in α-SMA immunoreactivity per µm^2^ of mural cells over time (Figure 6B), suggesting an increased contractility of the blood vessels during metastases development. Comparable to α-SMA, we observed a perivascular expression of MLCK (Figure 6C), known to be expressed by pericytes [32]. Semi-quantitative analysis of MLCK immunoreactivity revealed a sustained increase by exposure to malignant cells, especially in close vicinity to well-stablished metastases, from seven days onwards (Figure 6D). 

These results demonstrate that, during BCC extravasation, the expression of α-SMA and MLCK increased in mural cells, highlighting the involvement of smooth muscle cells and/or pericytes’ activation and contractility in BCBM formation. 

### 2.7. PLVAP Expression Occurs in BCBM

**Figure 7:**
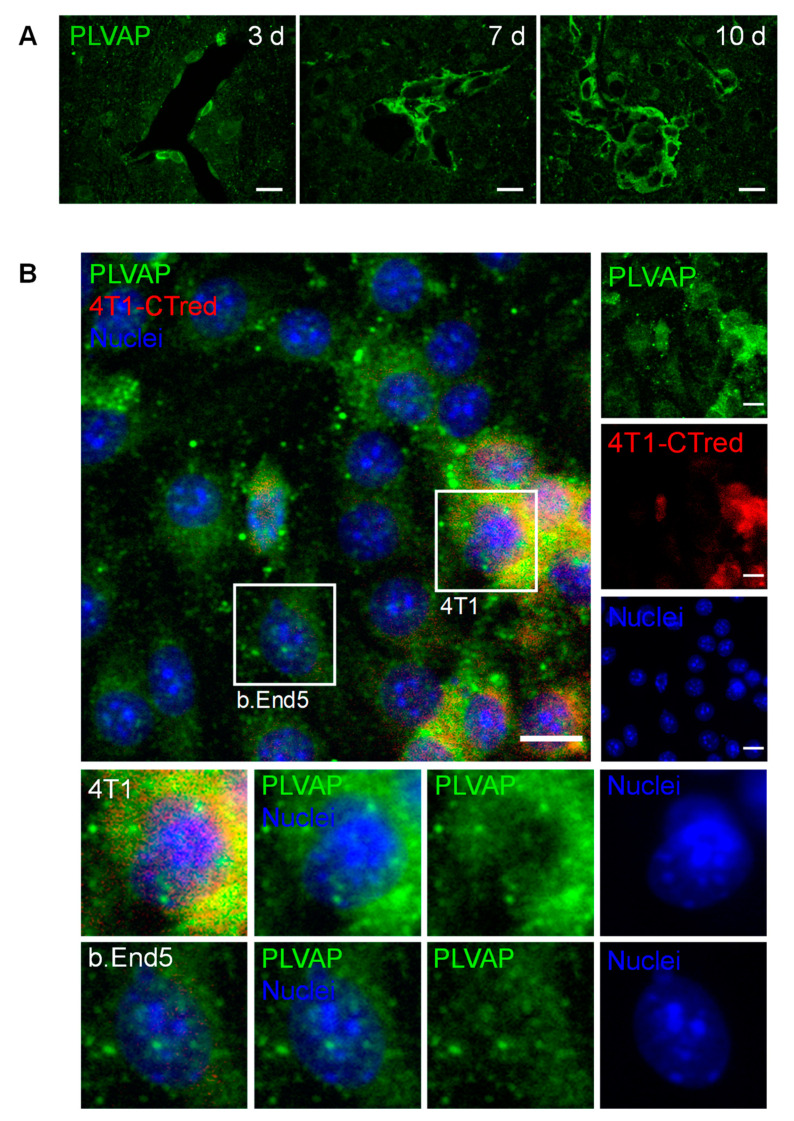
Plasmalemma vesicle-associated protein (PLVAP) expression occurs in breast cancer (BC) brain metastases formation. The 4T1 cells or vehicle (controls) were inoculated in the carotid arteries of female Balb/c mice and hippocampal sections were analyzed after 5 h (h), 3, 7 and 10 days (d). (**A**) Endothelial cells-specific protein PLVAP was observed in isolated metastatic BC cells and in well-established metastasis close to blood vessels. Scale bar: 20 µm (**B**) Triple negative BC cells in mixed culture with brain microvascular endothelial cells express PLVAP. Confluent monolayers of b.End5 cells (endothelioma cell line) were exposed to laminar non-pulsatile physiologic shear stress (1.5 dyn/cm^2^) achieved by orbital rotation for 24 h and then exposed to 4T1-cells labelled with CellTracker™ Red CMTPX (4T1-CTred, red), for distinction of both cell types, and a 24 h incubation was performed. Mixed cultures were immunolabelled for PLVAP (green). Magnified sections show PLVAP expression of each cell type. Nuclei (blue) were counterstained with Hoechst 33342. Scale bar: 10 µm.

The expression of PLVAP, a membrane EC-specific protein expressed in large tumors with implications in new vessel formation [23], was also inspected (Figure 7). Surprisingly, PLVAP staining was not only observed in isolated brain microvasculature, but also in BCBM, specially at the cell contour in well-established metastases (Figure 7A). To confirm this observation, immunolabelling for PLVAP was performed in mixed cultures of 4T1 TN BCCs and b.End5, a BMEC cell line, under shear stress to better mimic the in vivo conditions. Interestingly, a clear expression of this protein was observed not only in BMECs but also in BCCs (Figure 7B). To the best of our knowledge, this is the first study reporting the expression of PLVAP in non-ECs. 

Overall, these results point to the importance of new vessel formation in BCBM development and reveal PLVAP in malignant cells as a new actor.

## 3. Discussion

**Figure 8:**
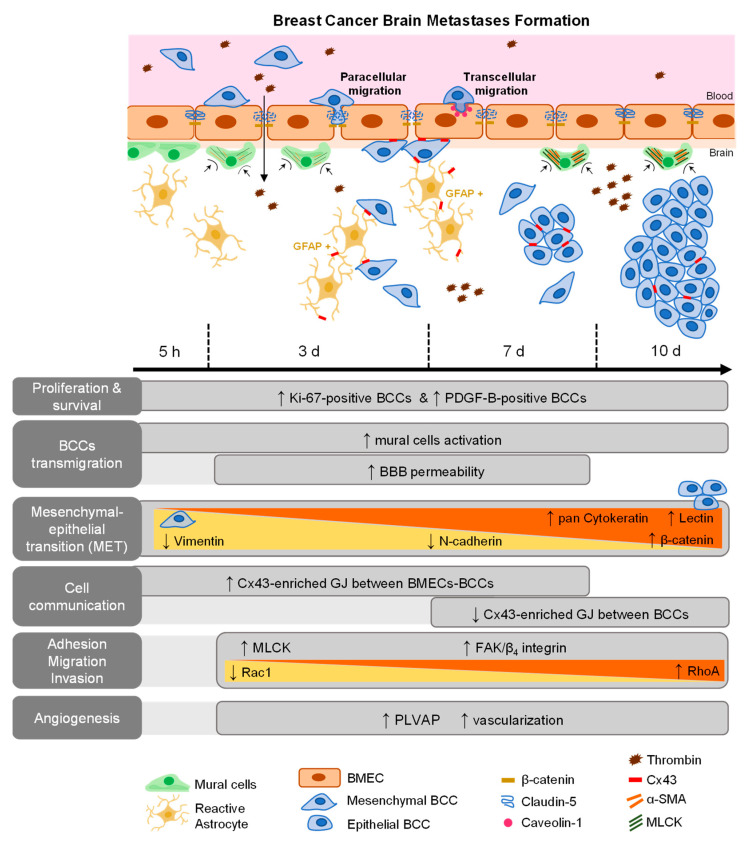
Schematic representation of major events involved in breast cancer (BC) brain metastases formation. BC cells (BCCs) entering brain parenchyma presented mesenchymal, migratory, and proliferative features; however, with time, they increasingly express epithelial markers, reflecting a mesenchymal–epithelial transition (MET). Malignant cells expressed platelet-derived growth factor B (PDGF-B), as well as β_4_ integrin and focal adhesion kinase (FAK), suggesting autocrine and/or paracrine regulation mechanisms with integrin and adhesion signaling activation. Decreasing Rac1 and increasing RhoA expression are in line with decreased motility with metastasis development. Gap junctions (GJ)’ formation was clear among tumor, between tumor and brain endothelial cells, or in astrocytes, highlighting intercellular communication role during disease progression. Concomitant BBB disruption, with junctional protein impairment and increased transcytosis support paracellular and transcellular alterations with extravasation. Plasmalemma vesicle-associated protein (PLVAP) expression by BCCs, together with increased vascularization, were also observed.

BCBM is an intricate and complex disease, which has been under-investigated despite its high incidence and poor patients’ outcome. It is believed that the interaction between metastatic cells and BMECs plays an important role in the formation of BCBM [18], involving BCCs adhesion to the brain vasculature, their transmigration, and tumor-associated vascular development [5,16,33]. However, the concerted events governing in vivo BCBM formation are still insufficiently understood. Using a robust and reproducible animal model of preferential formation of metastases in the brain [18,26,34], we disclosed the temporal profile of BCBM formation and relevant players involved in BCC extravasation. Moreover, we unveiled the role of adhesion and migration signaling pathways activation, MET, cellular crosstalk, mural cells’ activation, as well as routes of BBB hyperpermeability and increased vascularization towards a successful brain colonization (Figure 8). 

The detection of well-established BCBM seven days after BCCs inoculation corroborates our previous work [26]. Here, we further observed Ki-67-positive BCCs already at five hours, which is in line with our previous observations of intravascularly localized proliferating (EdU-positive) tumor cells before proceeding to extravasation [34]. At later timepoints, Ki-67-positive cells were especially seen in the border of the lesions, suggesting a higher proliferative capacity of these cells comparatively to the core of the metastasis. Ki-67 is expressed in the nucleus during different phases of the cell cycle [35], and was shown to be significantly higher in brain metastases than in the primary breast tumors, being prognostically discriminant for survival in BCBM patients with either HER2 overexpression or TNBC [35,36,37]. Accordingly, we observed an increase in Ki-67-positive TN BCCs over time, reflecting augmented cell proliferation and disease severity. 

Metastatic BCCs also exhibited PDGF-B expression, a growth factor described to support blood vessels’ maturity, functionality, and, consequently, tumor growth [6,38]. Its upregulation and pathway activation were shown to occur in highly invasive glioma cells [39], as well as during EMT and metastases formation [40]. Moreover, another study has identified PDGF-B expression as a prognostic marker for brain metastases, but not metastases to other sites [41]. In the present study, PDGF-B-positive BCCs were preferentially located in proximity to blood vessels, thus assuring proper oxygen and nutrient supplies, essential for their proliferation. Similarly, the observed expression of this growth factor may constitute a mechanism of autocrine and/or paracrine regulation of cell survival and proliferation [40,41], which highlights PDGF-B as a key player in the brain colonization of TNBC. Its receptor, PDGFRβ, is primarily expressed in pericytes; therefore, PDGF–PDGFR interaction might constitute another mechanism of the pro-metastatic effect of brain pericytes, in addition to the secretion of ECM proteins and insulin-like growth factor 2 [20].

Upregulation of mesenchymal markers such as vimentin has been associated to tumor progression and increased invasiveness [42]. Vimentin is an intermediate cytoskeleton filament protein, which contributes to cell motility through EMT-related transcription factor regulation [43]. BCCs entering brain parenchyma express vimentin, a feature that indicates a migratory and invasive phenotype. Another mesenchymal marker is N-cadherin, a transmembrane adhesion molecule normally found in neural, mesenchymal, and connective tissue cells, which is associated with invasion and migration processes and is highly expressed in human malignancies [44,45]. In initial timepoints, BCCs were N-cadherin-negative, becoming positive after extravasation [18]. We found that a decrease in the number of N-cadherin-positive metastatic cells occurs at the later stages (10 days) of BCBM formation, which was accompanied by the appearance of epithelial markers, such as tomato lectin, β-catenin, and cytokeratin. Indeed, BCCs already expressed β-catenin in early stages, an expression that was maintained throughout time with progressive positioning in the membrane. These observations indicate the reepithelization of BCCs, known as MET, a mechanism that may promote their adaption and survival in the brain microenvironment [4]. Interestingly, malignant cells that were positive only for cytokeratin occupied the core of the metastases, whereas vimentin-positive (whether negative or positive for cytokeratin) were positioned at the periphery, indicating the retaining of migratory/invasive mesenchymal characteristics of marginal BCCs. By expressing both markers, it appears that BCCs can maintain their phenotypic plasticity, suggesting that BCCs may undergo a total or partial MET accordingly with their needs, a phenomenon already observed in BCBM patients [46]. 

Besides MET, mechanisms of adhesion, invasion, and migration are also determinant for a successful secondary organ colonization [47]. Previous studies have demonstrated high FAK mRNA levels, as well as a higher FAK immunoreactivity in TNBC cells than in non-TNBC and in normal breast tissues [48,49]. Accordingly, our results revealed FAK expression in BMECs and metastatic BCCs, suggesting the activation of signaling pathways involved in tumor invasion and migration. In fact, during tumorigenesis, FAK increase is often associated with the upregulation of integrins [50]. Thus, we further analyzed the expression of β_4_ integrin, found in several cancers [8], and correlated with tumor progression and poor patient survival [51]. We found that BCCs co-express FAK and β_4_ integrin at advanced stages of tumorigenesis, suggesting the formation of FAK/β_4_ integrin complexes that may lead to downstream signaling activation [52] responsible for motility and cytoskeleton contraction. 

The equilibrium between Rac1-mediated membrane protrusion and RhoA-mediated contractility is crucial for the spatiotemporal coordination of cytoskeletal dynamics in moving cells [10], controlling the formation of focal adhesion complexes and lamellipodia, responsible for cell attachment and movement [53]. Indeed, Rac1 is linked with tumor cell migration, while RhoA is associated with tumor growth and metastasis formation [54,55]. Moreover, alterations in RhoA subcellular localization towards the nuclei in oxidative or inflammatory damage have been reported [55,56]. Therefore, the observed Rac1 decrease and RhoA increase in well-established metastases, with RhoA nuclear translocation at advanced stages, suggest decreased BCCs motility with metastasis enlargement and the existence of extensive brain damage. Interestingly, the increase in β_4_ integrin co-labelling with FAK (from 7 to 10 days) appears after the early expression of Rac1 (three days) and concomitant with the later decrease in Rac1 and increase in RhoA expression (from 7 to 10 days), reflecting the MET suffered by BCCs. Concomitant with such alterations was the observation of MLCK-positive BCCs three days after 4T1 cell injection, preferentially in the vicinity of blood vessels and later in well-established metastases, supporting the occurrence of cytoskeleton changes. MLCK’s role in tumor cell motility and invasiveness has been not only reported in vitro but also in lung cancer patients who showed disease recurrence and distant metastases [12,13]. Accordingly, the observed MLCK expression in extravasating BCCs appears to be determinant in cell motility, especially in BCCs close to microvessels. 

Analysis of Cx43 revealed its expression in the contact areas between BCCs and BMECs with an increasing intensity until seven days, an observation in line with the time range described for the BCC extravasation to the brain [57]. These findings suggest that Cx43 upregulation in tumor cell–EC contact areas may be involved in the attachment and extravasation processes, suggesting intercellular communication to be less necessary afterwards during tumor growth, as observed by the Cx43 decrease at 10 days. Accordingly, the study of lung metastases revealed a higher Cx43 expression in vessels containing cancer cells comparably with vessels with no malignant cells [58], and a reduction in metastasis formation by using a GJ inhibitor that decreased the communication between malignant cells and brain endothelium in a Cx43-dependent manner [59]. Our findings indicate that the formation of GJ enriched with Cx43 between BCCs and ECs could facilitate the intercellular communication required for BCCs adhesion and transmigration into the brain. Furthermore, they revealed an increase in Cx43 expression in BCCs in well-established metastases, which had never been described before in BCBM. Therefore, it appears that BCCs communicate between them and may transfer essential molecules through GJ channels in order to survive and grow after leaving the circulation. 

Cx43 is also relevant for other brain cells, being widely expressed in adult astrocytes and upregulated in reactive astrocytes [29,60]. Inflammation is associated with cancer initiation and progression by supplying bioactive molecules to the tumor microenvironment, including growth and survival factors that limit cell death [61]. At later stages, astrocytes share a bidirectional communication with tumor cells, helping to support tumor growth [62]. Likewise, we observed an increased colocalization between Cx43 and GFAP during BCC extravasation into the brain, mainly in non-peritumoral astrocytes This result suggests that, when BCCs colonize the brain, reactive astrocytes expressing Cx43 are recruited, which may promote BCCs proliferation and survival [63], via Cx43-mediated crosstalk.

By which pathways BCCs can extravasate into the brain is a question not yet fully clarified; it is unknown whether the passage of tumor cells leaves the BBB intact, and occurs by a paracellular route [16] or by the transcellular pathway [18]. We observed that TJs and AJs decreased expression, particularly in blood vessels with extravasating cells, pointing to an increased BBB paracellular permeability. Parallelly, an increase in blood-borne thrombin deposits was observed, notably in metastases-surrounding areas, suggesting BBB disruption during colonization of the brain by BCCs. Interestingly, at 10 days, a recovery of both β-catenin and claudin-5 immunoreactivity was observed, suggesting a possible reorganization of endothelial junctions and subsequent BBB integrity restoration. Caveolin-1 expression increased as BCBMs were formed, especially in BMECs feeding metastatic lesions. It is known that internalization mechanisms into BMECs can occur via caveolae, although it is still unclear whether caveolae comprise endocytic transport carriers themselves, or if they indirectly induce transcytosis through membrane fluidity modulation [64]. Furthermore, upon thrombin stimulation, it was shown that caveolin-1 leads to BBB junction reorganization and opening, determinant in barrier function [65]. Our current results indicate that the involvement of transcellular pathway activation in BCC extravasation cannot be discarded.

Pericytes have been widely studied in the context of their capacity to stabilize BBB properties and blood vessel structure, and their role towards brain metastasis development cannot be ignored. We have shown that pericytes play a crucial role in the development of metastatic brain tumors by secreting ECM proteins, which enhanced the adhesion of TN BCCs, and by secreting insulin-like growth factor 2, which had a pro-proliferative effect [20]. Alterations in mural cells contractility indicators during BCBM formation were observed, where both α-SMA and MLCK increased over time. The protein α-SMA is required for contraction within pericytes, explaining how blood vessels can change their diameter in response to environmental alterations [31]. This phenomenon may occur in our system, in which augmented mural cells’ marker expression was observed with metastatic development. Another possibility would be that exosomes secreted by BCCs might signal pericytes to become cancer-associated fibroblasts, which also express α-SMA, a mechanism observed in gastric cancer [66]. Besides α-SMA, Rho GTPases and Rho-associated protein kinase (ROCK) also regulate pericyte shape and increase contractility [32,67], in part by regulating the myosin light chain phosphorylation via MLCK, a protein whose expression increased in mural cells after seven days of BCC injections. As such, we may hypothesize that, at later stages, there is increased blood vessel contractility through actin and myosin modulation, in order to prevent the entrance of more BCCs into the brain parenchyma and as a mechanism to restore BBB impermeability (as observed by the increase in TJ and AJ at 10 days). 

By analyzing claudin-5 labelling, we observed an increased vascularization at seven days, which reflects BCCs’ necessity for oxygen and nutrient supplies for the development of larger brain metastases [17]. PLVAP is upregulated in ECs of well-vascularized tumors [68,69], which has been associated with increased tumor angiogenesis [23], whereas its downregulation prevented the development of pancreatic adenocarcinoma in xenografts [70]. Unexpectedly, we observed that PLVAP was clearly expressed by BCCs and in well-established BCBM, especially close to brain vessels. This observation was confirmed by the analysis of the protein expression in 4T1 cells in mixed cultures with BMECs. Interestingly, one report showed that quiescent metastatic melanoma cells in intravascular niches were negative for melanoma markers, and acquired EC features such as the expression of cluster of differentiation 31 (CD31) [71], a protein known to colocalize with PLVAP [23]. Therefore, our observations suggest that during BCBM formation, BCCs may acquire endothelial features that allow them to adapt and proliferate in the brain microenvironment, comprising a possible mechanism of cell transdifferentiation with clinical relevance. To the best of our knowledge, this is the first study reporting such phenomena in BCBM formation that deserve to be further studied.

Collectively, our work supports the evidence of known mechanisms in tumor progression but also provides a glimpse regarding earlier and late events in BCBM formation. Moreover, it sheds light onto new stages, such as BCC communication within lesions and cell transdifferentiation, as well as the involvement of PLVAP as a new actor. Nevertheless, our observations should be validated in the future using animal models taking advantage of brain-seeking BCCs, such as MDA-MB-231 BR, metastasizing with 100% frequency to the brain, and having progressively emerged as an established preclinical model of BCBM [72].

## 4. Materials and Methods

### 4.1. Cell Culture and Mouse Model of BCBM

A mouse model of BCBM, relying on the inoculation of murine mammary carcinoma TN 4T1 cells in the carotid arteries of Balb/c mice was used. The 4T1 cells were maintained in RPMI 1640 medium (PAN Biotech, Aidenbach, Germany) supplemented with ultra-glutamine I (Lonza, Basel, Switzerland) and 5% heat-inactivated fetal bovine serum (FBS, PAN Biotech) in a 5% CO_2_ atmosphere at 37 °C. BCCs (1 × 10^6^ 4T1 cells in 200 µL of Ringer-HEPES) were xenografted, under isoflurane anesthesia, in the right common carotid arteries of 7–8-week-old female Balb/c mice (Charles River Laboratories, Wilmington, MA, USA). Control mice were inoculated with Ringer-HEPES. Mice were housed and bred in the animal facility of the Biological Research Centre, Szeged, Hungary. Brains were harvested 5 h, 3 days, 7 days, and 10 days post-inoculation.

All animal experimentation was performed by certified team members at the Biological Research Centre, according to the recommendations of the Declaration of Helsinki and Tokyo and were performed according to the EU Directive 2010/63/EU on the protection of animals used for experimental and other scientific purposes. The protocol was reviewed and approved by the Regional Animal Health and Food Control Station of Csongrád County (license numbers: XVI./2980/2012 and XVI./764/2018). 

As an in vitro model that mimics the BCBM development, mixed cultures of mouse Balb/c brain endothelioma cell line, b.End5, and 4T1 cells were used. b.End5 cells were grown in Dulbecco’s modified Eagle’s medium (DMEM, Gibco, Life Technologies, New York, NY, USA) supplemented with 10% FBS, 1% non-essential amino acids (Biochrom AG, Berlin, Germany), 2 mM L-glutamine (Biochrom), 1mM sodium pyruvate (Biochrom) and 1% antibiotic-antimycotic solution (Sigma Aldrich, St. Louis, MO, USA). b.End5 cells (5 × 10^4^ cells/mL) were plated onto glass coverslips covered with rat tail collagen I (Corning, NY, USA) at 50 µg/mL, and after 48 h, laminar non-pulsatile physiologic shear stress (1.5 dyn/cm^2^) was applied for 24 h. In order to distinguish both cell populations, 4T1 cells were labelled with CellTracker™ Red CMTPX Dye (2.5 µM; Thermo Fisher Scientific, Waltham, MA, USA), and then plated (1 × 10^5^ cells/mL) in DMEM on top of b.End5 monolayers. Mixed cultures were kept on shear stress conditions for 24 h, timepoint after which cells were fixed with 4% (*w/v*) paraformaldehyde (PFA, Sigma-Aldrich, St. Louis, MO, USA) in phosphate-buffered saline (PBS) for 20 min at room temperature.

### 4.2. Collection of Brains

Anesthetized mice were perfused with PBS, followed by tissue fixation with 4% PFA in PBS. Brains were harvested, post-fixed overnight in 4% PFA in PBS at 4 °C and kept in PBS containing 0.1% sodium azide. Cranial hippocampus (coronal sections at −1.82 mm Bregma coordinate), the most affected brain region in terms of BCBM formation in this animal model [26], were paraffin embedded and cut into 4 μm-thick sections.

### 4.3. Hematoxylin-Eosin Staining, Immunohistochemistry and Immunofluorescence

Hippocampal tissue sections were subjected to hematoxylin-eosin (HE) staining, immunohistochemistry (IHC) analysis of PDGF-B (growth factor), or immunofluorescence (IF) of: Ki-67 (proliferation marker); N-cadherin and vimentin (mesenchymal markers); cytokeratin and tomato lectin (epithelial markers); FAK and β_4_ integrin (adhesion-associated proteins); Rac1 and RhoA (invasion and migration-associated proteins); MLCK (cytoskeleton remodeling-associated protein); α-SMA (pericyte marker); β-catenin, claudin-5 and Cx43 (AJ, TJ, and GJ markers, respectively); caveolin-1 (caveolae protein); GFAP (astrocyte marker); thrombin (BBB disruption indicator) and PLVAP (EC-specific protein). PLVAP was further assessed in mixed cultures of b.End5 and 4T1 cells.

Tissue sections were deparaffinized and rehydrated, and HE staining was performed [26]. For IHC analysis, inhibition of endogenous peroxidase (3% hydrogen peroxide solution), heat-mediated antigen recovery (10 mM citrate buffer, pH 6.0) and blocking (3% bovine serum albumin, BSA, containing 0.5% Triton X-100) were performed prior to incubation with the primary antibody, anti-PDGF-B (1:75); afterwards, sections were developed using SuperPicture™ Polymer Detection Kit (Invitrogen, Paisley, UK), and mounted with Quick-D Mounting Medium (Klinipath, Netherlands). For IF analysis of tissue sections, antigen retrieval, permeabilization and blocking conditions, as well as antibodies’ details and dilutions are summarized in Table 1. For IF analysis of fixed cells, a permeabilization with 0.5% Triton X-100 for 5 min followed by blocking with 3% BSA for 60 min at room temperature was performed. Cells were incubated overnight at 4 °C with anti-PLVAP (1:50) and thereafter with the corresponding secondary antibody, IgG-FITC (1:100) for 60 min at room temperature, in the dark. Both primary and secondary antibodies (indicated in Table 1) were diluted in corresponding blocking solutions. Nuclei were counterstained with Hoechst 33342 (1:1000, Thermo Fisher Scientific, Waltham, MA, USA) followed by mounting with SlowFade^®^ Diamond Antifade Mountant (Thermo Fisher Scientific, Waltham, MA, USA). Negative controls with the omission of primary antibodies were performed to exclude nonspecific binding or cross reactivity.

### 4.4. Image Acquisition

Images of HE and PDGF-B staining were acquired using an Olympus BX51 wide-field epifluorescence microscope coupled to a TIS DFK 1.9MP Sony CCD color camera. Images of IF staining of Ki-67, cytokeratin, vimentin, Rac1, MLCK, claudin-5, β-catenin, thrombin and PLVAP were acquired using a confocal microscope (Leica TCS SPE), equipped with 488, 532, and 635 nm lasers, a Leica DFC365 FX 1.4MP CCD camera and Leica HCX PL APO objectives. Images of α-SMA, N-cadherin, tomato lectin, Cx43, GFAP, FAK, β_4_ integrin, RhoA, and caveolin-1 staining were acquired using an epifluorescence microscope (Olympus BX60) equipped with an Olympus U-RFL-T Mercury lamp, a Hamamatsu Orca R2 cooled monochromatic CCD camera, and Olympus UPlanApo objectives. Ten fields per section at each timepoint were acquired and analyzed.

### 4.5. Data Analysis

Metastasis appearance and development were analyzed based on the evaluation of HE staining, where total tumor area was determined by the delimitation of each metastasis per field, and results expressed as tumor area (μm^2^). Rac1 and RhoA immunoreactivities in brain metastases were assessed based on the delimitation of each metastasis, measuring its mean intensity and normalizing by the area; for N-cadherin and Cx43, the mean intensity was measured and normalized by cell number. Cx43 immunoreactivity near blood vessels was also analyzed by measuring its mean intensity. Counts of the number of positive cells per field was performed for PDGF-B, Ki-67, vimentin (cells with round-shape morphology only), cytokeratin, FAK and β_4_ integrin staining. For claudin-5, β-catenin and caveolin-1, each blood vessel was delimited, the vascular immunoreactivity of the protein measured, and the results expressed by the mean intensity per μm^2^ of blood vessel; an equivalent approach for α-SMA and MLCK was performed, where the results were expressed by the mean intensity per μm^2^ of mural cell. For microvascular density evaluation, the area of claudin-5-positive blood vessels per field was measured [73], being expressed as the total area of blood vessels per μm^2^ of brain tissue. Thrombin entrance into the brain parenchyma [30] was performed by counting the total number of thrombin deposits per section. All the referred data analyses were conducted using ImageJ software 1.29x software (National Institutes of Health, USA). GJs in astrocytes were evaluated through colocalization between Cx43 and GFAP using Icy software (Institute Pasteur and France Bioimaging, France).

### 4.6. Statistical Analysis

Results obtained were analyzed using GraphPad Prism^®^ 6.0 (GraphPad Software, San Diego, CA, USA) and are expressed as mean ± SEM. One-way ANOVA and the Dunnett post hoc test were used to compare parameters evolution over time of 4T1 cell-injected mice, and between 4T1 cell-injected and vehicle-injected mice at each timepoint. *p*-values less than 0.05 were considered statistically significant.

## 5. Conclusions

Overall, our findings demonstrate that TN BCCs extravasation occurs early in time, inducing BBB and brain parenchyma alterations. PDGF-B appears to be an actor in early and sustained BCCs proliferation, with signaling cascades induced by β_4_ integrin/FAK signaling, and involving Rac1/RhoA, as well as MLCK, presenting a determinant role in BCCs adhesion, migration, and cytoskeleton contractility. The hub role of the GJ protein Cx43 in cell communication when metastases are established was observed, either among BCCs or with BMECs. BBB disruption with paracellular and transcellular pathways alterations may reflect BCC transmigration by both routes, whereas pericyte contraction indicates mural cells’ activation. Importantly, during brain colonization, BCCs undergo MET and transdifferentiation, namely by acquiring endothelial features such as PLVAP. By picturing the different steps, molecular players, and cellular crosstalk during the process of BCBM development, this study helps to solidify the current understanding and points to targets for modulation in order to counteract BCBM.

## Figures and Tables

**Table 1:** Summary of the experimental conditions and antibodies used for IF analysis.

Marker	Antigen Retrieval	Permeabilization	Blocking	Primary Antibody	Secondary Antibody
α-SMA	10 mM citrate buffer pH 6.0	0.5% Triton X-100	3% BSA + 0.5% Triton-X 100	α-SMA (1:100) Abcam, #ab5694, Rabbit Pc	Alexa Fluor^®^ 488 (1:500) Thermo Fisher Scientific, #A-11034, Goat anti-Rabbit
β-catenin	10 mM citrate buffer pH 6.0	0.5% Triton X-100	10% GS + 0.5% Triton-X 100	β-catenin (1:20) Thermo Fisher Scientific #71-2700, Rabbit Pc	Alexa Fluor^®^ 555 (1:100) Thermo Fisher Scientific, #A-21428, Goat anti-Rabbit
β_4_ integrin	10 mM citrate buffer pH 6.0	0.5% Triton X-100	3% BSA + 0.5% Triton-X 100	β_4_ integrin (1:250) Santa Cruz Biotechnology #SC-514426, Mouse Mc	Alexa Fluor^®^ 488 (1:500) Thermo Fisher Scientific, #A-11001, Goat anti-Mouse
Caveolin-1	10 mM citrate buffer pH 6.0	0.01% saponin	3% BSA + 0.01% saponin	Caveolin-1 (1:250) Cell Signalling #3238, Rabbit Pc	Alexa Fluor^®^ 555 (1:500) Thermo Fisher Scientific, #A-21428, Goat anti-Rabbit
Claudin-5	10 mM citrate buffer pH 6.0	0.5% Triton X-100	3% BSA + 0.5% Triton-X 100	Claudin-5 (1:500) Thermo Fisher Scientific #35-2500, Mouse Mc	Alexa Fluor^®^ 647 (1:500) Thermo Fisher Scientific, #A-21235, Goat anti-Mouse
Cx43	10 mM citrate buffer pH 6.0	0.5% Triton X-100	10% BSA + 0.5% Triton-X 100	Cx43 (1:50) Thermo Fisher Scientific #35-5000, Mouse Mc Cx43 (1:50) Abcam #ab11370, Rabbit Pc	Alexa Fluor^®^ 647 (1:500) Thermo Fisher Scientific, #A-21235, Goat anti-Mouse Alexa Fluor^®^ 555 (1:500) Thermo Fisher Scientific, #A-21428, Goat anti-Rabbit
FAK	10 mM citrate buffer pH 6.0	0.5% Triton X-100	3% BSA + 0.5% Triton-X 100	FAK (1:250) Abcam #ab131435, Rabbit Pc	Alexa Fluor^®^ 555 (1:500) Thermo Fisher Scientific, #A-21428 Goat anti-Rabbit
GFAP	10 mM citrate buffer pH 6.0	0.5% Triton X-100	3% BSA + 0.5% Triton-X 100	GFAP (1:100) Sigma-Aldrich #G3893, Mouse Mc	Alexa Fluor^®^ 488 (1:500) Thermo Fisher Scientific, #A-11001, Goat anti-Mouse
Ki-67	10 mM citrate buffer pH 6.0	0.5% Triton X-100	3% BSA + 0.5% Triton-X 100	Ki-67 (1:100) Thermo Fisher Scientific #PA5-19462, Rabbit	Alexa Fluor^®^ 555 (1:500) Thermo Fisher Scientific, #A-21428 Goat anti-Rabbit
MLCK	10 mM citrate buffer pH 6.0	0.5% Triton X-100	3% BSA + 0.5% Triton-X 100	MLCK (1:50) Thermo Fisher Scientific #PA5-15177, Rabbit Pc	Alexa Fluor^®^ 555 (1:500) Thermo Fisher Scientific, #A-21428, Goat anti-Rabbit
N-cadherin	10 mM citrate buffer pH 6.0	0.5% Triton X-100	3% BSA + 0.5% Triton-X 100	N-cadherin (1:100) Thermo Fisher Scientific #PA5-19486, Rabbit Pc	Alexa Fluor^®^ 555 (1:500) Thermo Fisher Scientific, #A-21428, Goat anti-Rabbit
Pan cytokeratin	10 mM citrate buffer pH 6.0	0.5% Triton X-100	3% BSA + 0.5% Triton-X 100	Pan Cytokeratin (1:100) Thermo Fisher Scientific #MA5-12231, Mouse Mc	Alexa Fluor^®^ 647 (1:500) Thermo Fisher Scientific, #A-21235, Goat anti-Mouse
PLVAP	10 mM citrate buffer pH 6.0	0.5% Triton X-100	3% BSA + 0.5% Triton-X 100	PLVAP (1:20) Santa Cruz Biotechnology #sc-50168, Goat Pc	IgG-FITC (1:100) Santa Cruz Biotechnology, #sc-2024, Donkey anti-Goat
Rac1	10 mM citrate buffer pH 6.0	0.5% Triton X-100	5% GS in 3% BSA + 0.5% Triton-X 100	Rac1 (1:25) Thermo Fisher Scientific #PA1-091, Rabbit Pc	Alexa Fluor^®^ 555 (1:100) Thermo Fisher Scientific, #A-21428, Goat anti-Rabbit
RhoA	10 mM Tris-borate EDTA buffer pH 8.0	0.5% Triton X-100	10% BSA + 0.5% Triton-X 100	RhoA (1:10) Thermo Fisher Scientific #OSR00266W, Rabbit Pc	Alexa Fluor^®^ 555 (1:100) Thermo Fisher Scientific, #A-21428, Goat anti-Rabbit
Thrombin	10 mM Tris-borate EDTA buffer pH 8.0	-	3% BSA	Thrombin (1:200) Santa Cruz Biotechnology #sc-271449, Mouse Mc	Alexa Fluor^®^ 647 (1:500) Thermo Fisher Scientific, #A-21235, Goat anti-Mouse
Tomato lectin	10 mM citrate buffer pH 6.0	0.5% Triton X-100	3%BSA + 0.5% Triton-X 100	-	Tomato lectin (1:500) Vector Laboratories #FL-1171
Vimentin	10 mM citrate buffer pH 6.0	0.5% Triton X-100	3%BSA + 0.5% Triton-X 100	Vimentin (1:100) Thermo Fisher Scientific #PA5-27231, Rabbit Pc	Alexa Fluor^®^ 555 (1:500) Thermo Fisher Scientific, #A-21428, Goat anti-Rabbit

α-SMA, α-smooth muscle actin; Cx43, connexin 43; BSA, bovine serum albumin; FAK, focal adhesion kinase; GFAP, glial fibrillary acidic protein; GS, goat serum; Mc, monoclonal; N-cadherin, neuronal cadherin; PLVAP, plasmalemma vesicle-associated protein; Pc, polyclonal; Rac1, Ras-related C3 botulinum toxin substrate 1; RhoA, Ras homolog gene family member A.

## Data Availability

The datasets used and/or analyzed during the current study are available from the corresponding author on reasonable request.
